# Artificial Intelligence in Orthopedic Surgery: Current Applications, Challenges, and Future Directions

**DOI:** 10.1002/mco2.70260

**Published:** 2025-06-25

**Authors:** Fei Han, Xiao Huang, Xin Wang, Yong‐feng Chen, Chuang Lu, Shasha Li, Lu Lu, Da‐Wei Zhang

**Affiliations:** ^1^ Department of Orthopedics Xijing Hospital Air Force Medical University Xi'an China; ^2^ Department of Orthopedics The 990th Hospital of the Joint Logistics Support Force Zhumadian China; ^3^ Lintong Rehabilitation and Convalescent Centre of the Joint Logistics Support Force Xi'an China

**Keywords:** artificial intelligence, deep learning, multimodal data fusion, orthopedic surgery, robot‐assisted surgery

## Abstract

Artificial intelligence (AI) drives transformative changes in orthopedic surgery, steering it toward precision and personalization through intelligent applications in preoperative planning, intraoperative assistance, and postoperative rehabilitation/monitoring. Breakthroughs in deep learning, robotics, and multimodal data fusion have enabled AI to demonstrate significant advantages. Nonetheless, current applications face challenges such as limited real‐time decision autonomy, fragmented medical data silos, standardization gaps restricting model generalization, and ethical/regulatory frameworks lagging behind technological advancements. Therefore, a critical analysis of the current status of AI and the acceleration of its clinical translation is urgently required. This study systematically reviews the core advancements, challenges, and future directions of AI in orthopedic surgery from technical, clinical, and ethical perspectives. It elaborates on the “perceptual‐decisional‐executional” intelligent closed loop formed by algorithmic innovation and hardware upgrades, summarizes AI applications across surgical continuum, analyzes ethical and regulatory challenges, and explores emerging trajectories. This review integrates the end‐to‐end applications of AI in orthopedics, illustrating its evolution. It introduces an “algorithm‐hardware‐ethics trinity” framework for technical translation, providing methodological guidance for interdisciplinary collaboration. Additionally, it evaluates the combined efficacy of diverse algorithms and devices through practical cases and details of future research frontiers, aiming to inform researchers of current landscapes and guide subsequent investigations.

## Graphical abstract

AI drives transformative changes in orthopedic surgery, steering it toward precision and personalization through intelligent applications in preoperative planning, intraoperative assistance, and postoperative rehabilitation/monitoring. Breakthroughs in deep learning, robotics, and multimodal data fusion have enabled AI to demonstrate significant advantages. Nonetheless, current applications face challenges such as limited real‐time decision autonomy (reliance on static preoperative data), fragmented medical data silos, standardization gaps restricting model generalization, and ethical/regulatory frameworks lagging behind technological advancements. Therefore, a critical analysis of the current status of AI and the acceleration of its clinical translation is urgently required. This study systematically reviews the core advancements, challenges, and future directions of AI in orthopedic surgery from technical, clinical, and ethical perspectives. It elaborates on the “perceptual‐decisional‐executional” intelligent closed loop formed by algorithmic innovation and hardware upgrades, summarizes AI applications across the full surgical continuum, analyzes ethical and regulatory challenges, and explores emerging trajectories. This review integrates the end‐to‐end applications of AI in orthopedics, illustrating its evolution from assistive to semi‐autonomous systems. It introduces an “algorithm‐hardware‐ethics trinity” framework for technical translation, providing methodological guidance for interdisciplinary collaboration. Additionally, it evaluates the combined efficacy of diverse algorithms and devices through practical cases and details of future research frontiers, aiming to inform researchers of current landscapes and guide subsequent investigations.

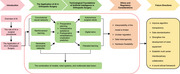

## 1 Introduction

The origins of artificial intelligence (AI) can be traced back to the early philosophical explorations of mechanical thinking and intelligent machines, as proposed by thinkers such as Descartes and Leibniz, who provided the theoretical foundation and inspiration for later AI research. However, the groundwork for modern AI was not established until the development of computers in the 1940s and the pivotal theoretical contributions of Alan Turing in the 1950s that the groundwork for modern AI was truly established [1]. The term “artificial intelligence” was formally introduced at the Dartmouth Conference in 1956, an event widely recognized as the official starting point of AI research [1, 2, 3]. Since then, AI has undergone several transformative phases, from rule‐based expert systems to deep learning, evolving into a powerful tool capable of autonomous decision making based on predefined rules and learned data patterns, thereby enhancing efficiency and addressing complex problems [4, 5]. In the 21st century, the exponential growth of data and the emergence of deep learning technologies have propelled AI into a new era. Breakthroughs in methods such as convolutional neural networks (CNNs) [6, 7, 8] and generative adversarial networks (GANs) [9, 10, 11] have laid the foundation for integrating AI into medicine. In particular, CNNs have dominated computer vision and medical imaging, often surpassing human performance in key tasks. Their ability to autonomously learn and generate representative feature subsets from sufficiently large training datasets significantly expands the scope and potential of AI applications in medicine [12]. By stacking multiple convolutional layers, CNNs progressively form abstract and complex hierarchical representations. In recent years, pathology image interpretation has evolved from manual expert analysis to machine learning and, more recently, to deep learning approaches. While expert systems rely on rule sets defined by specialists and traditional machine learning depends on handcrafted features, CNNs can learn directly from raw data and efficiently produce accurate outputs. This capability has led to the rapid adoption of CNNs in medical domains, especially imaging, where their superiority in pattern recognition and image analysis has enabled AI to gain substantial traction [12, 13, 14]. Moreover, the integration of reinforcement learning algorithms and continuous development of novel deep learning architectures have markedly enhanced the adaptability of AI in dynamic environments, offering theoretical support for real‐time decision‐making in surgical settings [15, 16, 17, 18]. Today, AI has emerged not only as an advanced computational tool but also as a transformative paradigm comprising diverse algorithmic systems designed to solve real‐world problems and fulfill practical functions. By simulating human reasoning to optimize outcomes and boost efficiency, AI is reshaping the landscape of modern medicine and ushering in a new model for technological integration into healthcare [19, 20, 21].

The earliest and most prominent demand for AI in medicine emerged in the fields of image analysis and diagnostics. For instance, AI applications in lung cancer screening [22, 23] and breast cancer detection [24, 25] have not only improved diagnostic accuracy but also significantly reduced reporting turnaround times [26]. In addition, AI has demonstrated considerable potential in expediting pathological analysis [27, 28], accelerating novel drug discovery [29, 30], and supporting the development of personalized multimodal treatment strategies [31, 32]. These advancements have substantially enhanced clinical efficiency and reduced healthcare costs, offering valuable insights into the deeper integration of AI within orthopedic surgery [15]. With the continual increase in computational power, rapid advancements in cloud computing, and the relentless innovation and optimization of task‐specific algorithms by technical experts, AI systems are becoming increasingly integral to orthopedic surgical practice [33, 34]. Despite the field's inherent challenges—such as complex anatomical structures and the demand for high‐precision operations—AI is rapidly reshaping traditional surgical paradigms, particularly in areas like preoperative planning, intraoperative navigation, and postoperative recovery management [35, 36, 37, 38]. Orthopedic procedures are characterized by high anatomical complexity and many interventions depend heavily on the surgeon's expertise and real‐time judgment. However, the reliance on human skills is inherently constrained by cognitive and physical limitations. AI, empowered by data‐driven algorithms and high‐throughput computation, offers more objective, reproducible, and evidence‐based support for surgical decision‐making across the entire perioperative continuum, from planning and execution to rehabilitation [39, 40, 41, 42]. For example, by leveraging deep learning to analyze patient imaging data, AI can rapidly construct three‐dimensional reconstructions, accurately localize fracture sites, predict optimal surgical approaches, and generate personalized postoperative rehabilitation plans. These capabilities aid clinicians in decision‐making while providing detailed, individualized guidance for patient recovery. Consequently, AI reduces preoperative preparation time, enhances surgical precision, minimizes operative risk and duration, and ultimately promotes optimal functional restoration, making it a key direction in the future evolution of orthopedic surgery [43, 44].

In recent years, the application of AI in surgical procedures has expanded rapidly and is emerging as a pivotal force driving innovation in surgical practice. Robotic‐assisted systems such as Mako [45] and da Vinci [46] have been widely adopted in orthopedic surgery, leveraging real‐time data processing and complex trajectory planning to dynamically adapt to surgical requirements. These platforms have significantly improved prosthesis alignment accuracy and reduced postoperative recovery time [47]. Furthermore, AI‐powered real‐time navigation and augmented reality (AR) technologies have demonstrated remarkable precision in spinal surgery, offering dynamic support for complex procedures [48, 49, 50]. Although AI has made substantial strides in orthopedic surgery, particularly in imaging‐based diagnostics and preoperative planning, several critical challenges persist. These include limited autonomy in real‐time navigation and minimally invasive procedures as well as barriers to clinical integration and scalability. Notably, issues such as fragmented medical data systems, lack of standardized data formats, and ongoing ethical and legal scrutiny surrounding AI‐generated decisions remain major hurdles to widespread implementation [51, 52, 53].

This review aimed to systematically examine the current state and future prospects of AI in orthopedic surgery. We focused on key technological advances and persistent challenges in three primary domains: preoperative planning, intraoperative navigation, and postoperative rehabilitation. By synthesizing existing evidence and analyzing emerging trends, this work seeks to provide comprehensive guidance for both researchers and clinicians and to offer theoretical insights that may support the continued evolution of AI applications in orthopedic surgery.

## 2 Applications of AI in Orthopedic Surgery

With the rapid advancement of AI technology and its increasing application in orthopedic surgery, significant clinical value has been demonstrated across various aspects, including preoperative planning, intraoperative assistance, postoperative rehabilitation, and patient care. This progress has propelled the continuous development of precision medicine models. Specifically, the shift from traditional preoperative surgical planning to personalized plan designs and multidimensional simulation optimization has led to more accurate treatment strategies for surgeons. Real‐time navigation and dynamic decision support have enhanced surgical precision, markedly reduced the workload, and decreased the complexity and difficulty of surgeries. Moreover, targeted management and monitoring of complications, along with personalized rehabilitation training programs, have transformed postoperative monitoring and rehabilitation paradigms in orthopedics. AI‐integrated multimodal data models offer patient‐tailored monitoring and rehabilitation plans, whereas remote AI guidance has introduced new treatment paradigms. Consequently, further application of AI in orthopedic surgery is poised to significantly alter current medical models.

### 2.1 Preoperative Planning

#### 2.1.1 Hot Applications of Medical Image Analysis

**FIGURE 1:**
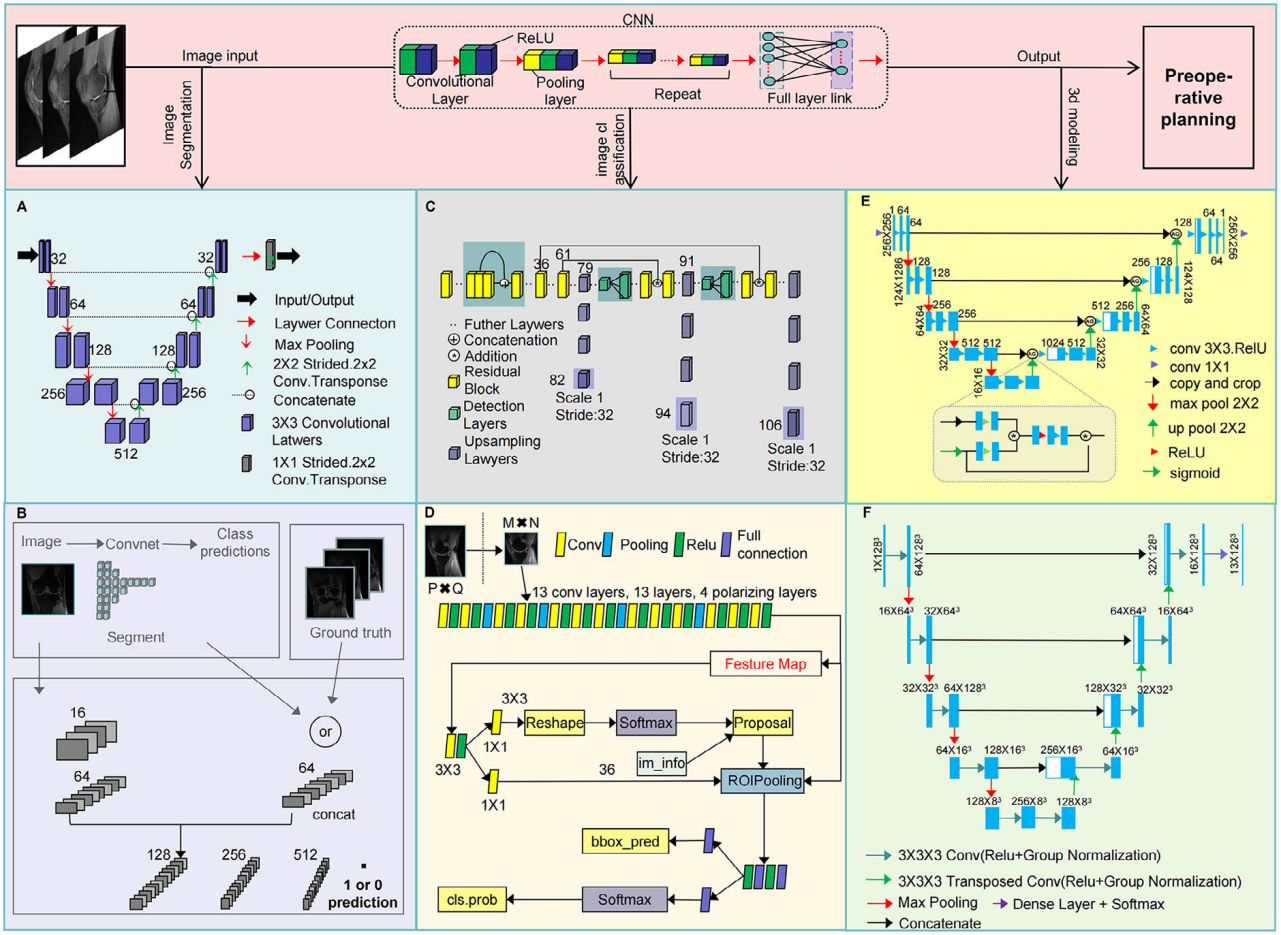
Key applications of CNN‐based AI in preoperative imaging analysis for orthopedic surgery. (A) The U‐Net model utilizes an encoder–decoder symmetric structure with skip connections to achieve high‐precision segmentation of orthopedic imaging data. (B) The GAN model enhances the usability and analytical precision of preoperative orthopedic imaging through an adversarial training mechanism between the generator and discriminator. (C) The YOLO model, with its single‐stage object detection architecture and multiscale feature fusion capability, improves the accuracy and efficiency of orthopedic preoperative image classification. (D) The Faster R‐CNN model integrates a region proposal network and a multitask loss function for object detection, enabling efficient localization of bony anatomical structures, precise identification of pathological regions, and end‐to‐end training optimization for classification performance. (E) The Enhanced Attention Res‐UNet model combines ResNet, U‐Net architecture, and an attention mechanism to achieve high‐precision segmentation and 3D reconstruction of medical images. (F) The 3D U‐Net model integrates 3D convolution operations, an encoder–decoder structure, skip connections, and multiscale feature fusion to perform high‐precision voxel‐wise segmentation of 3D medical images.

The earliest and most significant applications of AI in preoperative planning for orthopedic surgery were centered on medical imaging analysis and processing. As a foundational element in surgical planning, the quality and interpretability of imaging data directly influence the diagnostic accuracy and the formulation of operative strategies [54, 55]. In recent years, AI‐driven deep learning techniques, particularly CNNs, have significantly advanced medical image processing with groundbreaking progress in image segmentation, classification, and three‐dimensional (3D) modeling (Figure 1) [56, 57, 58].

Traditional imaging interpretation relies heavily on physician experience and often requires manual annotation of key anatomical structures, which is time consuming and prone to human bias [59, 60]. However, the integration of AI, particularly CNN‐based models, has significantly enhanced the automation of image segmentation. Notably, the Double U‐Net model, which consists of two cascaded U‐Net networks, achieves high‐precision orthopedic segmentation and complex lesion identification (Figure 1A) [61, 62]. In addition, GAN‐based segmentation models enable cross‐modal image synthesis and feature alignment, effectively resolving issues related to ambiguous orthopedic margins. These models also facilitate small‐sample data augmentation and annotation optimization, thereby addressing the problem of limited medical datasets. Furthermore, adversarial training enhances segmentation robustness and improves model adaptability to noise and artifacts (Figure 1B) [63, 64]. Collectively, these advances have made automated image segmentation a reality, allowing AI to integrate and analyze high‐resolution imaging data, thereby detecting subtle details beyond human perception and improving diagnostic accuracy [65, 66].

Image classification is another key advancement in preoperative planning. Conventionally, tasks such as lesion localization require a high level of expertise, and complex fractures or tumors may be misidentified. Deep learning‐based detection algorithms such as You Only Looking Once [67] and Faster R‐CNN [68] can rapidly scan medical images and precisely localize abnormal regions (Figure 1C,D). CNN‐based classifiers further refine this process by integrating vast amounts of physician expertise, digitizing radiographic information, and incorporating patient‐specific clinical data to enhance diagnostic accuracy while significantly reducing workload, time consumption, and misdiagnosis rates [69, 70]. For example, in bone tumor detection, AI‐powered deep learning models not only distinguish benign from malignant tumors but also predict the extent of tumor invasion, providing critical insights for preoperative decision‐making [71].

Beyond image classification, AI‐driven 3D modeling technology offers more intuitive anatomical visualization for preoperative planning. AI‐based reconstruction of computed tomography (CT) or magnetic resonance imaging (MRI) data allows for the generation of detailed 3D models of patient‐specific anatomy. The Enhanced Attention Res‐UNet model, which integrates residual network (ResNet), U‐Net architecture, and an attention mechanism, significantly improves the accuracy of bone structure segmentation, optimizes complex anatomical feature extraction, and enables the creation of high‐fidelity 3D models for surgical planning (Figure 1E) [72]. Furthermore, the 3D U‐Net model demonstrates remarkable precision in segmenting bony structures, optimizing prosthesis matching, and assisting surgical navigation, thereby enhancing orthopedic surgical accuracy and efficiency (Figure 1F) [73, 74]. These technological advancements address traditional limitations in 3D modeling by improving accuracy and capturing detailed anatomical variations, helping surgeons to evaluate pathological regions from multiple perspectives and develop more precise surgical strategies [75, 76]. For instance, AI‐driven 3D modeling in spinal deformity correction surgery can precisely measure vertebral rotation angles and curvature deviations, reducing errors to within 0.5°, optimizing surgical path planning, and significantly improving physician–patient communication efficiency [76].

Despite the remarkable progress in AI in imaging‐based preoperative planning, its full potential is yet to be realized. Saravi and colleagues [77], for example, highlighted the use of hybrid deep learning models that incorporate genomic, radiological, and clinical data, paving the way for multi‐input architectures that synthesize diverse patient‐specific information. Therefore, multimodal data integration, interdisciplinary collaboration, and equitable AI implementation have become active areas of research. These approaches promise to provide more comprehensive and precise preoperative insights, reduce surgical complexity and risk, and ultimately shorten the operative time [78, 79].

#### 2.1.2 Applications of AI‐Based Surgical Planning Models

Another pivotal area in preoperative planning is the development and implementation of AI‐based surgical planning models. These models integrate patient‐specific clinical data with imaging analysis to generate precise and individualized surgical strategies, predict postoperative outcomes, streamline orthopedic workflows, reduce costs, and enhance operational efficiency [80, 81]. In recent years, the broad application of AI technologies has significantly improved the scientific rigor and practicality of surgical planning, with notable benefits in areas such as path optimization and virtual simulation [82, 83]. For example, Chen et al. [2] utilized over 1.2 million CT images from 3000 patients to develop an AI‐powered preoperative planning (AIHIP) system for total hip arthroplasty (THA). In a prospective validation study involving 120 patients, the AIHIP system demonstrated superior accuracy, imaging outcomes, and clinical performance compared with traditional planning methods. This significantly reduces the time and manpower required to formulate a detailed surgical plan [84]. In a systematic review of studies indexed in PubMed, Scopus, and Web of Science, Mozafari et al. analyzed six investigations encompassing 831 THA patients and found that AI‐assisted preoperative planning significantly improved femoral component positioning accuracy [54]. In particular, AI has demonstrated superior performance in both femoral and acetabular alignments, supporting its value as an indispensable tool for optimizing THA outcomes [85, 86]. These findings underscore the growing importance of AI as a powerful tool for surgical planning.

The integration of virtual reality (VR) and AR technologies with AI further enhances preoperative planning capabilities. Using AI‐generated 3D anatomical models, surgeons can simulate procedures within a virtual environment prior to the actual operation [43]. This form of “virtual surgery” training enables physicians to familiarize themselves with patient‐specific anatomical details and identify potential risk zones, thereby increasing surgical success rates, minimizing intraoperative complications, reducing operative time, and lowering healthcare costs [80, 87]. These benefits are particularly evident in complex procedures, such as joint replacement [88], bone tumor resection [89], and spinal surgeries [90], where VR and AR are emerging as transformative tools. As their adoption continues to expand, these technologies are expected to redefine operative planning and intraoperative navigation, paving the way for a new paradigm in orthopedic surgery [90].

Another critical function of AI‐based surgical planning models is the optimization of surgical pathways. Traditionally, surgical route planning has relied heavily on the expertise and prior experience of surgeons. In contrast, AI can automatically refine and optimize surgical paths using machine learning algorithms and subsequently assist surgeons in achieving greater precision in intraoperative execution and decision‐making [91]. This is particularly valuable in orthopedic oncology, where the integration of AI with robotic systems enables more accurate surgical strategies and optimized pathways, thereby reducing procedural complexity. Such advancements are increasingly applied in bone tumor surgeries [92, 93].

To further explore this trend, we reviewed the applications of AI surgical planning models in orthopedic procedures published over the past 3 years, as summarized in Table 1. The analysis revealed that, as AI models continue to evolve and robotic technologies mature, their functional capabilities and scope of application in surgical planning have expanded significantly. These models are capable of addressing increasingly complex surgical challenges. Cui et al. [94] developed a multicenter interactive AI platform to predict postoperative functional decline in patients with metastatic spinal disease, thereby assisting clinicians with surgical planning decisions. Their work highlights the transformative potential of AI as a surgical planning tool [94]. Looking ahead, AI‐driven surgical platforms are expected to integrate a wider array of cutting‐edge technologies. The adoption of such innovations holds promise for overcoming data silos in healthcare systems and enabling collaborative data sharing across institutions. This will further enhance the generalizability, accuracy, and clinical utility of AI as a central component in surgical planning.

**TABLE 1:** Applications, advantages, and limitations of AI models in preoperative planning for orthopedic surgery.

AI model	Applications	Advantages	Limitations
3D U‐Net [95]	Fully automated 3D spinal CT reconstruction and pedicle screw trajectory planning	High segmentation accuracy; supports personalized screw diameter and length recommendation	Dependent on high‐quality CT data; sensitive to low‐resolution imaging
Graph neural network [96, 97]	Fracture biomechanics pathway modeling and optimization of reduction strategies	Dynamically simulates mechanical distribution; recommends most stable reduction plans	High computational complexity; requires high‐performance GPU support
ResNet‐50 + Attention [98, 99]	Prosthesis size prediction and alignment correction planning	Low prediction error for implant sizing; accommodates anatomical variations in Asian populations	Requires real‐time calibration with intraoperative navigation
Generative adversarial network [100]	Generate synthetic MRI data for preoperative training and protocol simulation	Addresses small sample size issues; high diversity of generated data	Synthetic images may lack realistic pathological details
Deep reinforcement learning [101, 102]	Robot assisted continuous path planning for pedicle screws	Optimizes screw insertion angles in real time; reduces risk of neural injury	Requires extensive surgical video data for training; generalizability remains unproven
XGBoost + CNN fusion [103, 104]	Prediction of dislocation risk and prosthesis positioning in total hip arthroplasty	Integrates imaging and clinical data	Limited model interpretability; reliant on structured electronic health records
AutoSeg‐Mask R‐CNN [18]	Intervertebral disc degeneration segmentation and spinal fusion planning	High segmentation accuracy; supports fusion cage size recommendation	Poor performance in segmenting calcified tissues
Multitask learning [105]	Simultaneously plan the angle of the acetabular cup and bone preservation strategy (accurately and quickly design acetabular cup prostheses)	Reduces intermodel coordination error; shortens planning time	Requires multicenter validation for generalizability
Surgical transformers [106, 107]	Surgical video‐based preoperative planning and intraoperative decision support	Captures long‐range temporal dependencies; supports dynamic plan adjustment	Requires specialized hardware for accelerated inference
Federated learning [108]	Multicenter collaborative training for joint alignment planning in arthroplasty	Preserves data privacy; improves model generalization	High communication cost; slow convergence
Swin transformer + reinforcement learning [109, 110, 111]	Robot path planning for minimally invasive scoliosis correction	Adaptive to tissue deformation; high obstacle‐avoidance success rate	Requires calibration of force feedback devices
Multimodal foundation model [47, 54]	Multiobjective optimization in total hip arthroplasty	Integrates X‐ray, bone density, and gait data; accurate prediction of dislocation risk	Requires input of multiple clinical parameters
Bayesian deep learning [112, 113]	Dynamic evaluation of personalized surgical risk	Quantifies uncertainty; enables confidence visualization	Requires Markov Chain Monte Carlo sampling

Abbreviations: CNN, convolutional neural network; XGBoost, extreme gradient boosting.

### 2.2 Intraoperative Assistance

Surgical procedures often require surgeons to make rapid and irreversible decisions under highly individualized patient conditions. These decisions rely heavily on the surgeon's expertise, clinical experience, and real‐time judgment, thereby introducing a potential human bias. Therefore, the need for intelligent systems that can provide timely information and decision support has become increasingly pressing in orthopedic surgery. AI, particularly CNNs, has demonstrated substantial potential for intraoperative applications, particularly in real‐time data processing, robotic control, and dynamic surgical strategy adjustment. By guiding surgeons through complex intraoperative workflows, AI significantly enhances the precision and safety of surgical procedures. Moreover, with the growing adoption of surgical robotics, AI has played an essential role in enabling faster, more efficient, and safer surgeries, thereby contributing to improved patient outcomes [114, 115].

Real‐time data acquisition and processing are fundamental for precise surgical execution. The involvement of AI at this stage significantly enhances image fusion and anatomical structure recognition. By integrating intraoperative imaging modalities such as CT, MRI, and ultrasound with preoperative 3D models, AI systems can dynamically update anatomical visualizations, offering surgeons high‐resolution and real‐time navigation to support expert decision‐making [116, 117]. For example, in a study by Wang et al. [118] on robotic navigation in spinal surgery, current systems incorporated intraoperative imaging (e.g., CT and radiography) with preoperative plans to generate dynamic anatomical reconstructions. These platforms, which are powered by precise image registration algorithms, enable surgeons to perform minimally invasive spinal procedures using robotic assistance. Additionally, ongoing algorithmic innovations continue to reduce the margin of error in pedicle screw placement, thereby improving procedural efficiency and enhancing real‐time anatomical interpretation [118].

The convergence of AI with advanced robotic technologies has ushered orthopedic surgery into a new era of intraoperative precision. In particular, the role of AI in real‐time image processing and adaptive surgical path adjustment has expanded the capabilities of surgical robots, helping to overcome the limitations of manual procedures and suboptimal ergonomics. By analyzing intraoperative imaging data, AI systems can improve navigation accuracy and surgical safety [119]. This technology also reduces the need for repeated image acquisition, which can disrupt the surgical workflow, and allows lead surgeons to dynamically adjust strategies based on real‐time AI feedback. As a result, surgical accuracy and therapeutic efficacy are significantly enhanced, which stems from the continuous progress and integration of AI and robotic technologies [120, 121].

Table 2 summarizes recent (past 3 years) applications of AI models in orthopedic surgery, illustrating the transformative impact of AI on intraoperative practices. Notably, the development of multimodal data fusion has emerged as a research hotspot, enabling automated and personalized surgical planning to guide intraoperative decision‐making [122]. By combining intraoperative imaging with real‐time biomechanical data, AI platforms are becoming increasingly capable of delivering comprehensive surgical support, thereby optimizing the precision and success rates of complex orthopedic procedures.

**TABLE 2:** Recent applications of AI models in intraoperative assistance for orthopedic surgery (past 3 years).

**AI model**	Applications	Advantages	Limitations
CNN for automatic landmark extraction [123, 124, 125]	Anatomical landmark recognition during robot‐assisted joint replacement (e.g., femoral condyles, acetabular key points)	Fast extraction speed, low error rate, reduces manual point selection errors	Depends on high‐quality CT data; manual calibration required for cases with bone defects
Two‐stage multitask deep learning framework [105]	Pelvic bone segmentation and landmark detection (for patients with developmental dysplasia of the hip)	Enhanced segmentation accuracy for abnormal skeletal structures; reduced landmark localization error	Requires dedicated GPU computation; limited intraoperative real‐time capability
AI‐hip system (deep CNN) [126, 127]	Intelligent decision‐making for acetabular cup safe zones in total hip arthroplasty	Integrates pelvic neutral position algorithms; low angular error in cup placement	Requires intraoperative navigation validation; limited suitability for patients with severe osteoporosis
Deep stacked network + hybrid prior knowledge [128]	Dynamic prediction of acetabular safe zones and intraoperative adjustment	High prediction accuracy; supports intraoperative biomechanical feedback	Requires fusion of preoperative CT with intraoperative pressure sensor data
Remote collaborative AI algorithm [129]	Real‐time navigation for robot‐assisted fracture reduction (tremor filtering, visual enhancement)	Low localization error; supports interregional collaboration	Relies on 5G network coverage; signal stability may be affected by metal implants
Mako robotic arm‐assisted TKA system [130]	Dynamic optimization of force lines during knee replacement surgery (based on multi center mechanical data)	The intraoperative force line error is small, and the prediction of prosthesis life is accurate and high	Requires standardized data annotation; model update cycle is relatively long
3D intelligent registration algorithm [131]	Real time 3D navigation during surgery (for robot fracture surgery navigation)	Fast real‐time feedback and high accuracy	Requires manual calibration in regions covered by osteophytes
Reinforcement learning‐based robotic arm control algorithm [132, 133]	Adaptive obstacle avoidance during spinal deformity correction (dynamic tissue deformation compensation)	High success rate of obstacle avoidance; reduces risk of nerve injury	Requires calibration of force feedback devices; high computational resource consumption
AI‐finite element hybrid simulation model [134, 135]	Real‐time prediction of implant stress distribution and optimization of osteotomy volume	Low error in biomechanical simulation; high osteotomy efficiency	Requires real‐time CT scanning during surgery to update model input
Optical tracking + deep learning fusion algorithm [136, 137]	Real time tracking and navigation of percutaneous screw placement for thoracolumbar vertebral fractures during surgery	Low angular deviation in screw placement; exceeds accuracy limits of manual techniques	Requires intraoperative O‐arm scanning for severe spinal deformities
GAN [138]	Imaging data synthesis and surgical simulation	Solves small sample problem; high diversity of generated data	Synthetic images may lack realistic pathological details
ResNet‐50 + attention mechanism [99, 139]	Prediction of joint replacement prosthesis size and force line correction	Low prediction error; adapts to anatomical variations in Asian populations	Requires real‐time calibration via intraoperative navigation
XGBoost [140, 141]	Automatic detection of bone metastases in CT imaging	High sensitivity; satisfactory positive predictive value	Requires large volumes of annotated training data
AI + 3D‐printed guide plate integrated system [142, 143]	Complex joint replacement (e.g., cases with “stiff knee”)	Solves challenges posed by severe osteophyte encapsulation; reduces surgical time and clinical cost	Requires dedicated 3D printers; limited accessibility in primary care institutions
Dynamic joint adaptation algorithm [144, 145]	Personalized prosthesis design and control of leg length discrepancy	Error controlled within millimeter range; excellent prosthesis conformity	Requires intraoperative navigation for patients with congenital skeletal deformities

Abbreviations: CNN, convolutional neural network; GAN, generative adversarial network; TKA, total knee arthroplasty; XGBoost, extreme gradient boosting.

### 2.3 Postoperative Complication Prediction and Rehabilitation Guidance

#### 2.3.1 Applications of AI in Predicting and Managing Postoperative Complications

Effective management of postoperative complications is essential for the recovery of orthopedic patients. Traditional approaches often rely on the clinical experience of healthcare providers, which may be inefficient and lack personalization. In contrast, AI reshapes postoperative management by integrating multimodal data, enabling automated analysis and supporting intelligent decision making, thereby demonstrating significant potential in this domain [146, 147]. Recent studies have increasingly focused on AI models trained on large‐scale patient datasets to identify the risks of complications, such as postoperative infections and prosthetic loosening. These models provide robust support for precise and individualized clinical decisions [148, 149, 150]. AI has been applied to various aspects of postoperative care, including automated imaging analysis, clinical decision‐support systems, and remote patient monitoring. These technologies contribute to improved efficiency in postoperative workflows and facilitate the early detection of complications and personalized nursing strategies [151, 152].

Another emerging research focus is the use of AI to predict hospital readmission risk following orthopedic surgery. A previous study reported that AI achieved moderate predictive accuracy, with a mean C‐statistic of 0.71, whereas the best performance was observed in models targeting hip and knee arthroplasty, with a mean C‐statistic of 0.79 [153]. Although AI models are advantageous for their ability to comprehensively integrate preoperative, intraoperative, and postoperative data, current studies still face issues such as poor data standardization and methodological heterogeneity, which may introduce bias. Future research should focus on developing standardized protocols and enhancing data processing methodologies to yield more reliable and clinically applicable results, thereby promoting the broader clinical adoption of AI technologies [154, 155].

Recent AI models developed for postoperative complication prediction and management in orthopedic surgery are summarized in Table 3. Although early outcomes have been encouraging, several challenges remain, including concerns regarding data quality, model interpretability, and clinical integration [156]. We believe that future research should prioritize the clinical optimization of AI algorithms, focus on effective multimodal data fusion, and promote the advancement of personalized postoperative care strategies.

**TABLE 3:** Recent AI models for postoperative complication prediction and management in orthopedic surgery.

AI model	Applications	Advantages	Limitations
Multimodal model [157]	Postoperative infection risk prediction	Integrates large‐scale imaging data with nearly 100 algorithms; high predictive accuracy; supports multimodal fusion (imaging + text)	Relies on standardized annotation protocols; requires regular clinical database updates
Convolutional neural network [158]	Bone‐muscle modeling based on full‐length lower limb radiographs to predict joint loosening and gait abnormalities	Fully automated modeling; enhanced skeletal precision	Only supports static image analysis; sensitive to low‐resolution X‐rays
Deep learning (AIJOINT®3D surgical simulation) [159, 160, 161]	Prediction of prosthesis loosening and infection risk; evaluation of postoperative functional recovery	Covers the entire process from preoperative planning to intraoperative navigation and postoperative assessment; certified as Class III innovative medical device	Long update cycle (requires multicenter validation); sensitive to metallic artifacts
Machine learning (random forest + XGBoost) [162, 163]	Deep vein thrombosis (DVT) risk stratification; prediction of postoperative pain trends	Incorporates dynamic physiological parameters (e.g., tourniquet time)	Requires manual calibration of biomechanical parameters; does not integrate real‐time monitoring data
Federated learning model [164]	Multicenter collaborative training to optimize prognostic models (e.g., complication prediction)	Preserves patient privacy; improves model generalizability	High communication cost; requires unified data annotation standards
Multimodal large language models [165]	Full‐cycle complication management (pre‐, intra‐, and postoperative), integrating imaging, biomechanics, and EHR data	Cross‐modal correlation analysis; supports real‐time parameter adjustment	High deployment complexity; requires standardized data interface protocols
Blockchain + AI data management platform [166, 167]	Tracking medication adherence and tracing complications after surgery	Enables full‐process traceability of drug distribution; high accuracy in tracing prosthesis failure	Requires hospitals to join blockchain networks; high initial deployment costs
Generative adversarial network [168]	Prediction of joint range of motion after total hip arthroplasty	Dynamically simulates degrees of freedom; low prediction error	Requires dedicated sensor devices; low adoption rate in primary hospitals

Abbreviations: XGBoost: extreme gradient boosting.

#### 2.3.2 Role of AI in Personalized Rehabilitation Programs

With rapid advancements in AI and robotics, assistive robotic systems have emerged as promising innovations in rehabilitation, offering enhanced engagement and improved outcomes across diverse environments. AI algorithms enable dynamic data analysis, real‐time exercise guidance, and personalized feedback, thereby addressing the limitations of conventional postoperative rehabilitation such as insufficient customization and inconsistent adherence. These technologies facilitate precise training regimens and long‐term rehabilitation strategies, thereby significantly improving postoperative recovery outcomes [169, 170, 171]. Cha et al. [172] investigated the role of AI in postoperative sleep monitoring in patients with hip fractures. By analyzing sleep data, the AI system identified abnormal sleep patterns and provided tailored rehabilitation protocols to alleviate chronic pain and mitigate functional decline, which are critical to overall recovery [172]. These advancements underscore the transformative role of AI in orthopedic rehabilitation, particularly through the integration of gait analysis, imaging technologies, and VR.

**FIGURE 2:**
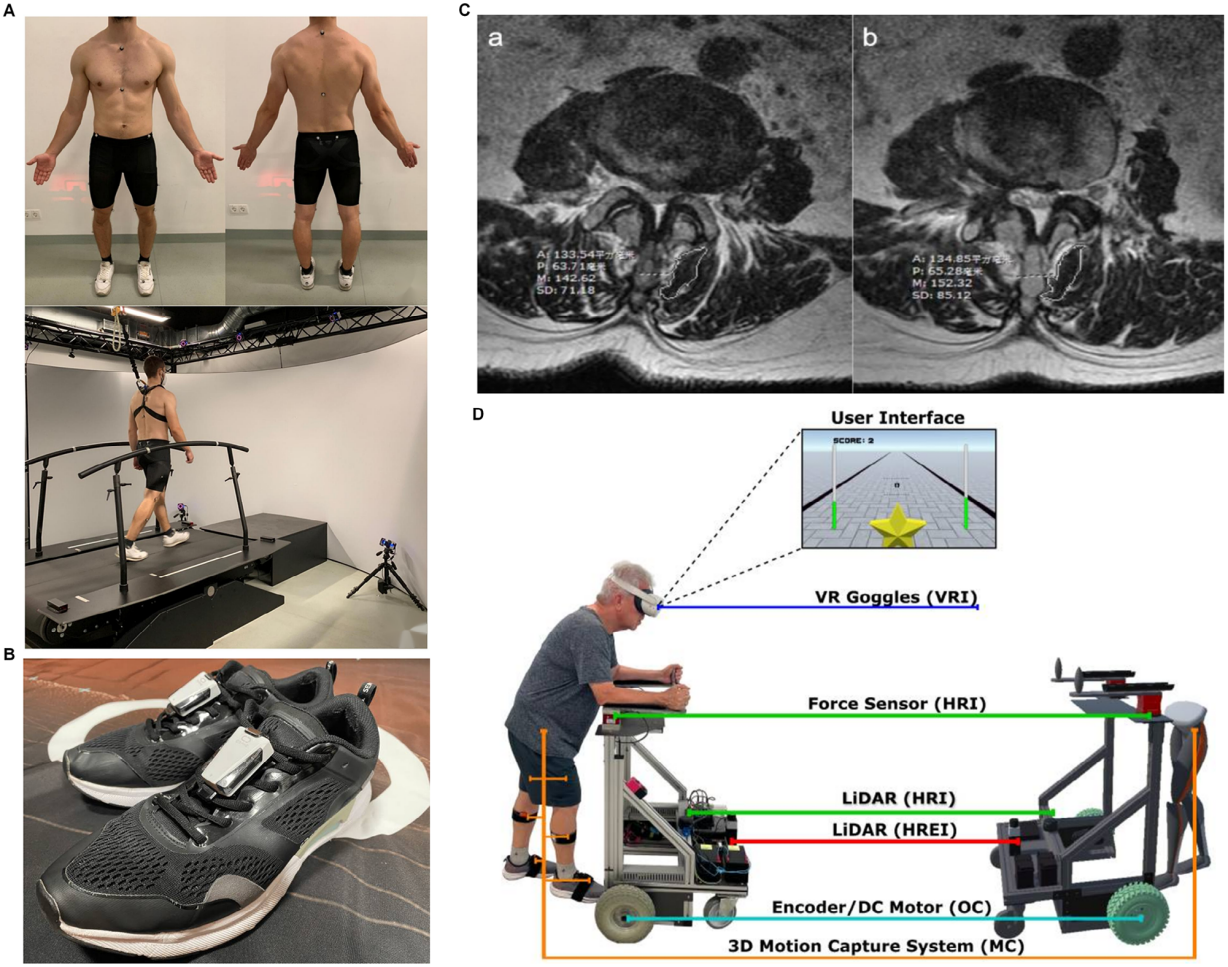
The role of AI in personalized rehabilitation programs. (A) Gait analysis dataset generation through self‐selected speed treadmill walking to determine 3D kinematic and dynamic changes. Adapted with permission [177]. Copyright © 2025, The Author(s): Stephan Oehme et al. (B) Gait data collection via wearable six‐axis sensors attached to both feet. Adapted with permission [178]. Copyright © 2025, Yoshikawa et al. (C) Axial T2‐weighted MRI analysis of lumbar muscle cross‐sectional area (CSA) using Philips MRI. Key parameters include A (CSA of the multifidus muscle in mm^2^), *P* (perimeter of the scribed area in mm), *M* (average gray value of the scribed area), and SD (standard deviation of the gray value). Adapted with permission [175]. Copyright © 2021, The Author(s): Zhen Lyu et al. (D) A participant using the immersive virtual reality scenario robot that integrates five subsystems: odometry and control (OC), human–robot‐environment interaction (HREI), human–robot interaction (HRI), motion capture (MC), and virtual reality integration (VRI). Adapted with permission [186]. Copyright © 2024, by the authors: Matheus Loureiro et al.

Gait analysis is a cornerstone of rehabilitation assessment and serves as a crucial basis for clinical decision‐making and refinement of individualized rehabilitation plans. With the advent of more efficient data‐transmission technologies and next‐generation AI algorithms, gait monitoring is no longer confined to hospitals. The COVID‐19 pandemic has accelerated the development of remote rehabilitation technologies and computational models [173, 174, 175]. Recent studies have compared AI‐guided remote rehabilitation for anterior cruciate ligament reconstruction with in‐hospital face‐to‐face rehabilitation training. After 6 months of follow‐up, no significant differences were observed between the two groups, and after 1 year, AI‐guided home‐based rehabilitation outperformed in‐hospital rehabilitation in terms of functional recovery. These findings suggest that AI‐based remote rehabilitation is highly effective, with gait analysis playing a crucial role in improving the assessment efficiency and accuracy. AI‐assisted gait monitoring and analysis can predict the recovery speed and potential functional impairments, allowing timely intervention and personalized rehabilitation plans [176]. Several AI‐integrated gait analysis systems have already been introduced into clinical practice. For example, self‐selected speed‐gait analysis systems use infrared cameras to track a set of 26 reflective markers, enabling 3D kinematic and kinetic assessments to evaluate the efficacy of autologous bone grafting combined with matrix‐induced autologous chondrocyte implantation for knee osteochondral defects, thereby optimizing rehabilitation strategies (Figure 2A) [177]. Additionally, wearable six‐axis inertial sensors (white devices) attached to both shoes captured the stride speed and bilateral parameters, including the heel‐strike angle, toe‐off angle, pronation angle, foot progression angle, vertical height, swing width, and stride length normalized to height. These parameters were used to assess gait alterations following total ankle replacement and guide rehabilitation adjustments to enhance postoperative functional recovery (Figure 2B) [178]. Thus, AI‐driven gait analysis revolutionizes rehabilitation by overcoming geographical and institutional barriers, enabling real‐time postoperative gait assessment and optimizing personalized rehabilitation strategies.

AI‐based imaging analysis plays a pivotal role in personalized rehabilitation, particularly in orthopedics where medical imaging is indispensable for evaluating musculoskeletal conditions. AI‐enhanced imaging techniques are now a key focus in postoperative orthopedic rehabilitation research [179, 180]. AI‐driven rehabilitation monitoring using medical imaging has shown promising results in clinical practice. For example, in postpercutaneous endoscopic lumbar discectomy rehabilitation, researchers have utilized lumbar MRI to assess the effectiveness of dynamic lumbar chain training by calculating the cross‐sectional areas of multiple lumbar muscles to evaluate rehabilitation progress. Moreover, the MyoMotion 3D motion capture and analysis system has been employed for gait analysis, integrating both imaging and biomechanical data to refine the rehabilitation plans, resulting in highly personalized and precise rehabilitation strategies (Figure 2C) [175]. The integration of AI‐based imaging analysis with gait assessment further enhances customization and real‐time adaptability of rehabilitation programs, ultimately improving patient outcomes.

The combination of AI, VR, and AR has introduced a novel paradigm for rehabilitation training. The development of wearable multidimensional motion sensors has enabled accurate recognition of subtle free‐motion patterns while simultaneously capturing real‐time biomechanical data from multiple body regions, opening new frontiers in postoperative orthopedic rehabilitation [181, 182, 183]. Several studies have demonstrated the ability of AI to monitor patient performance in VR‐based rehabilitation in real‐time and dynamically adjust training difficulty levels. This adaptive system enhances rehabilitation efficiency while minimizing the risk of overtraining‐related injuries, thereby improving the accessibility, efficiency, and overall effectiveness of orthopedic rehabilitation services [184, 185]. For instance, an immersive VR scenario robot integrates gait parameters, intelligent‐walker‐assisted gait, and VR‐assisted gait rehabilitation. Bilateral lower limb kinematic parameters were captured using a 3D wearable motion capture system, which also incorporated cybersickness symptom assessments via patient questionnaires. This AI‐enhanced VR system optimized rehabilitation protocols by improving neuromuscular control, independence, and locomotion recovery (Figure 2D) [186].

Despite its unique advantages and effectiveness, AI‐assisted rehabilitation faces significant challenges including high technological complexity and costly hardware requirements, which currently limit the widespread adoption of AI‐integrated VR and AR technologies. However, given its transformative potential, the integration of rehabilitation robotics with gait analysis, imaging analysis, and VR/AR technologies represents the future of postoperative orthopedic rehabilitation. This multimodal AI‐driven approach is poised to establish a new standard for high‐quality personalized medical rehabilitation.

## 3 Technological Foundations of AI in Orthopedic Surgery

Through the collaborative innovation of algorithm research and hardware devices, AI in orthopedic surgery is propelling the field toward greater precision and intelligence. The development and optimization of algorithms are the core driving forces behind multimodal data integration, offering orthopedic surgeons personalized and intelligent support during and after surgery. This facilitates precise diagnosis and treatment in clinical orthopedic practice. In addition, improved data processing and integration capabilities are progressively eliminating the data silo effect caused by disparate devices and various healthcare institutions. Being crucial for implementing AI technologies, hardware device upgrades continuously construct and refine the “perceptual‐decisional‐executional” intelligent closed‐loop system. Through deep integration with algorithms, these advancements have resulted in intelligent diagnostic and treatment systems that comprehensively cover continuous and holistic treatment protocols throughout the preoperative, intraoperative, and postoperative phases.

### 3.1 Algorithm Development and Optimization

Algorithm development is a core challenge in the application of computational methods to healthcare, particularly in the era of big data. Traditional statistical and machine‐learning techniques often struggle to accommodate the integration of multiple models and complex datasets, thereby failing to meet the demands of modern clinical practice. In contrast, AI offers a promising solution through the continuous optimization and innovation of algorithms [187]. In orthopedic surgery, the diversity and heterogeneity of clinical data pose significant obstacles to AI implementation. Orthopedic datasets typically encompass a wide range of modalities including preoperative imaging (e.g., radiography, CT, and MRI), intraoperative sensor‐acquired physiological data (e.g., mechanical load and hemodynamic signals), and postoperative monitoring information (e.g., gait analysis and rehabilitation assessments). These data types differ in their format, resolution, and temporal characteristics, making the development of robust data integration algorithms a central task in AI model construction. Algorithmic optimization, such as effective multimodal data fusion, not only enhances model predictive accuracy, but also delivers more comprehensive clinical decision support for orthopedic surgeons. Such advances have improved the precision and effectiveness of surgical planning, execution, and follow‐up care [188, 189, 190]. To better understand the current landscape, we summarized recent algorithmic applications in orthopedic surgery over the past 3 years, highlighting their strengths and limitations in Table 4. This overview provides a foundational reference for researchers and developers working at the intersection of algorithmic science and surgical innovation.

**TABLE 4:** Recent algorithmic models applied in orthopedic surgery: applications, strengths, and limitations.

Algorithm type	Application scenarios	Advantages	Limitations
Deep learning [191, 192, 193]	Fracture detection and classification, bone tumor identification for surgical guidance	Support complex fracture classification and tumor boundary recognition, integrate multiple information to guide surgical operations	Relies on high‐quality annotated data; generalizability limited by dataset diversity
Convolutional neural network [194, 195]	Image segmentation and disease diagnosis are used to guide orthopedic surgery	Automatically segmenting bone, cartilage, meniscus and other tissues, incorporating multiple tissues into the AI model to reduce the subjectivity of disease diagnosis evaluation	Requires high‐resolution imaging; high computational resource consumption
Decision trees and ensemble learning [195, 196]	Fracture diagnosis at various stages, bone metastasis detection, postoperative risk assessment	Works with fewer samples; high sensitivity and specificity	Sensitive to image quality; requires manual feature engineering
Reinforcement learning [197, 198]	Surgical path planning, dynamic parameter tuning, surgical training	Dynamically optimizes surgical plans; reduces intraoperative errors; simulates realistic surgery	Complex to train; requires large amounts of real‐time data
3D modeling and simulation algorithms [199, 200]	Preoperative planning, prosthesis design, intraoperative visualization, communication, and teaching	Sub‐millimeter precision; improves surgical accuracy; 3D visualization enhances efficiency	Dependent on high‐quality imaging; time‐consuming modeling
Real‐time navigation algorithms [201, 202]	Robot‐assisted surgery	High robotic arm accuracy; reduces radiation exposure	High cost; requires specialized personnel
Supervised machine learning [203, 204, 205]	Postoperative complication prediction, prosthesis longevity evaluation	Outperforms traditional comorbidity indices in prediction accuracy	Low model interpretability; depends on complete clinical data
Natural language processing [206, 207, 208]	Surgical plan generation and doctor–patient communication	Enhances automated report generation and communication	Relies on structured data input; limited multilingual support
Generative adversarial networks [100, 209]	Image enhancement, simulation training, intraoperative navigation	Generates high‐quality synthetic images; reduces reliance on real data	Synthetic images may lack pathological diversity
Image classification algorithms [210, 211, 212]	Lesion recognition and surgical planning support	Rapid lesion classification; aids early diagnosis and surgical decisions	Sensitive to image quality; requires multimodal data fusion
Object detection algorithms (YOLO/SSD) [213, 214, 215]	Pre/intraoperative lesion localization	Real‐time lesion detection; enhances surgical and diagnostic efficiency	Lower accuracy for small targets; requires high computational power
Image segmentation algorithms (e.g., U‐Net) [216, 217, 218]	Accurate segmentation of bone and soft tissues	Sub‐pixel segmentation accuracy; assists in surgical navigation	Requires large labeled datasets; computationally intensive
Vibration analysis algorithms [219, 220]	Monitoring during bone cutting	Real‐time detection of vibrations; avoids adjacent tissue damage	Needs high‐precision sensors; sensitive to environmental interference
5G remote control algorithms [221, 222]	Remote surgical collaboration	Supports ultra‐low latency operation; overcomes geographic limitations	Dependent on stable network environment; high security requirements
Transfer learning [139, 211]	Prosthesis model adaptation	Reduces labeling demand; enhances model generalizability	Performance may be affected by domain mismatch with pre‐trained models
Federated learning [223, 224]	Multicenter collaborative research; breaking data silos	Protects patient privacy; enables data integration	High communication overhead; slow convergence
Meta‐learning [225, 226]	Rapid adaptation to new surgical scenarios	Learns new tasks from few samples	Complex training process; requires many meta‐tasks
Discrete marching cubes algorithm [227, 228]	3D reconstruction and surgical simulation	Preserves topology; reduces triangle mesh count; improves real‐time interaction	Depends on high‐quality imaging; ambiguity resolution needed
Multimodal AI systems [188, 229]	Integration of imaging and clinical data	Comprehensive surgical assistance throughout the procedure; improves accuracy	Requires external validation; complex data fusion
XGBoost [230, 231, 232, 233]	Detection of postoperative complications (prosthesis loosening, infection) and bone metastasis	High sensitivity and specificity with small datasets	Requires manual feature engineering; sensitive to image noise
Automated osteotomy control algorithms [234, 235, 236]	Robot‐assisted joint replacement	Sub‐millimeter cutting accuracy; reduces manual errors	Depends on preoperative CT planning; high equipment cost
Spatial registration and calibration algorithms [237, 238, 239]	Robotic navigation systems	Achieves globally optimal matching through 6D transformation; overcomes limitations of ICP	Requires high‐resolution images; computationally intensive

Abbreviations: SSD: Single Shot MultiBox Detector; YOLO, You Only Look Once; 5G remote control algorithms: fifth‐generation mobile networks remote control algorithms; multimodal AI systems: multimodal artificial intelligence systems; XGBoost: extreme gradient boosting.

Building upon the data summarized in Table 4, it is evident that the development and performance of AI foundation models are highly dependent on both the quality and volume of the training data. In reality, inconsistencies in the data generated by various medical devices and across different hospital systems, such as nonstandardized formats and incomplete datasets, have become major obstacles to the implementation of large‐scale AI models in orthopedic surgery. Therefore, the development of standardized and generalizable algorithms tailored to orthopedic data is of paramount importance for the construction of future AI models. AI‐driven approaches to data cleaning and format transformation can facilitate uniform processing across multiple centers. Moreover, the role of AI in multimodal data integration and quality enhancement is gradually overcoming long‐standing bottlenecks in orthopedic surgical data processing. By dynamically merging electronic health records (EHRs) with real‐time intraoperative and postoperative data streams, AI systems can offer seamless support across the entire surgical continuum, from preoperative planning and intraoperative navigation to postoperative rehabilitation [240, 241, 242, 243]. However, the development of these algorithms remains an area of ongoing progress. As research increasingly focuses on artifact reduction, data harmonization, and robust multimodal feature extraction, the technological potential of AI in orthopedic surgery is expected to be unlocked [244].

### 3.2 Hardware‐Driven Applications of AI in Orthopedic Surgery

Hardware systems serve as the physical infrastructure that translates AI technologies into actionable capabilities in orthopedic surgical practice. Their role is particularly indispensable in real‐time data processing, precise operative execution, and fusion of multimodal information. Orthopedic surgical robots, intelligent navigation platforms, and high‐precision sensors, when deeply integrated with AI algorithms, form a closed‐loop system of “perception–decision–execution.” This synergy has significantly enhanced the standardization, reproducibility, and safety of orthopedic procedures [38, 245]. With the ongoing development of smart hospitals and digitally integrated operating environments, a growing number of surgical robots are being deployed in orthopedic operating rooms. The functionality of these devices depends not only on core components such as GPU‐accelerated computing units and high‐fidelity force feedback sensors, but also on the adoption of federated learning frameworks that allow secure and decentralized data sharing across institutions while protecting patient privacy. These systems enable multi‐institutional collaboration in model training without compromising data security or integrity [221]. To further clarify the current status of hardware implementation in this field, we have summarized recent advancements in orthopedic surgical hardware in Table 5, highlighting their specific clinical applications, technical strengths, and current limitations. This summary can serve as a practical reference for researchers and developers working on next‐generation intelligent orthopedic surgical systems.

**TABLE 5:** Recent applications of AI‐integrated hardware devices in orthopedic surgery: use cases, advantages, and limitations.

Device type	Application scenarios	Core technology/algorithm	Advantage	Disadvantage
Intelligent orthopedic surgical simulator [246, 247]	Surgical skill training for orthopedic surgeons	VR + reinforcement learning feedback	High accuracy in real‐time performance scoring; shortens training cycles	High hardware cost; software content updates depend on vendor
AI‐assisted microsurgical orthopedic assistant [248, 249]	Microvascular anastomosis and nerve repair	Optical zoom control + object detection	Automatically identifies vessels/nerves; improves success rate of anastomosis	Requires specialized microscopic instruments; limited compatibility
Intelligent orthopedic hemostasis energy platform [250, 251]	Precision coagulation during surgery	Impedance sensing + dynamic power regulation algorithm	Reduces intraoperative bleeding; minimizes thermal damage area	Sensitive to tissue moisture; requires frequent calibration
Targeted orthopedic nanorobots [252]	Targeted intraoperative drug delivery	Magnetic navigation control + AI path optimization	Increases local drug concentration; reduces systemic toxicity	Still in clinical trial phase; extremely high usage cost
Intelligent orthopedic rehabilitation system [253, 254]	Postoperative joint function recovery	Motion capture (Kinect) + reinforcement learning incentive mechanism	Improves patient compliance; shortens rehabilitation duration	Requires high‐performance GPU; difficult for elderly patients to operate
Orthopedic surgical voice control hub [255]	Intraoperative device control	Natural language processing (BERT) + voiceprint recognition	High instruction response accuracy; supports multilingual input	Recognition accuracy drops in noisy environments
Intelligent assisted joint replacement system [256]	Assistance in joint replacement procedures	Flexible electrodes + anomaly detection algorithm	Monitors lumbar flexion and energy expenditure per minute in real‐time; reduces surgeon fatigue and increases efficiency	Requires further validation for musculoskeletal pain and strain reduction in surgeons
AI‐powered orthopedic materials R&D platform [257, 258]	Development of new implant materials	GAN + molecular dynamics simulation	Shortens material development cycle	Requires interdisciplinary collaboration; high computational resource demand
Intelligent orthopedic waste processor [259]	Intraoperative medical waste sorting and disposal	Visual recognition (ResNet‐152) + robotic arm sorting	High classification accuracy; improves handling of infectious waste	Limited processing capacity; requires regular maintenance

Abbreviations: VR, virtual reality; motion capture (Kinect) + reinforcement learning incentive mechanism: kinect‐based motion capture‐augmented reinforcement learning with incentive mechanisms; natural language processing (BERT) + voiceprint recognition: bidirectional encoder representations from transformers (BERT) augmented multimodal voiceprint‐text processing system; AI‐powered orthopedic materials R&D platform: artificial intelligence‐powered orthopedic materials research and development platform; GAN: generative adversarial networks

Based on the analysis of the device types summarized in Table 5, it is evident that hardware‐level AI applications in orthopedic surgery remain in the early stages. Currently, most AI‐integrated devices are limited to specific procedural steps, rather than functioning as components of a continuous, unified system. Moreover, many of these devices are only capable of assisting in technically simple tasks, whereas core surgical procedures rely heavily on the skills and expertise of the operating surgeon. The high equipment cost, limited technological diversity, and steep learning curves further constrain the widespread adoption of AI in orthopedic surgery [250, 260].

### 3.3 Challenges in Algorithm Optimization and Hardware Advancement

AI algorithms provide a strong technical foundation for intelligent support in orthopedic surgeries. However, their clinical application has been hindered by several technical bottlenecks. One of the most critical issues is the “black‐box” nature of AI models, which leads to poor interpretability, thereby limiting their clinical trustworthiness and adoption [261, 262]. In orthopedic procedures, surgeons must understand the rationale behind AI‐generated predictions to validate the reliability of surgical path recommendations or risk assessments. However, the lack of algorithmic transparency, combined with the complexity of AI technologies, often hampers the clinicians' ability to fully grasp the underlying logic, reduces clinical confidence, and limiting real‐world effectiveness [263, 264, 265]. In recent years, progress in explainable AI has helped mitigate this issue. Tools such as heatmap visualizations allow clinicians to observe how AI models identify key anatomical structures or determine optimal surgical approaches, fostering greater trust in algorithmic outputs [266, 267]. Nonetheless, future research should focus on tailoring AI algorithms to the specific demands of orthopedic surgery, enhancing interdisciplinary collaboration, and improving both model transparency and clinical acceptance.

As the physical carriers of AI applications, hardware devices play a crucial role in translating algorithmic outputs into clinical actions. The reliability and performance of surgical robots, intelligent navigation systems, and sensor platforms directly affect their clinical utility. However, significant barriers remain in the hardware development. First, the economic tension between high equipment costs and long‐term maintenance impedes its widespread deployment, particularly in primary care settings. The need for specialized companies to handle repairs, updates, and calibrations further amplifies this burden [268]. Second, the lack of standardized interfaces for heterogeneous data limits the efficiency of multimodal data fusion. In orthopedic surgeries, disparate data formats among imaging systems, EHRs, and biomechanical sensors force developers to build custom data‐conversion modules for each device. This not only increases the system complexity and development timelines but also introduces potential data transmission errors [269]. Furthermore, delays in real‐time biomechanical feedback algorithms hinder surgical precision. Although some orthopedic robots use generative AI for 3D skeletal modeling, their compensation algorithms for soft tissue deformation still rely on static preoperative data and cannot fully adapt to intraoperative dynamic load changes, resulting in suboptimal feedback during surgery [270, 271]. In addition, the lack of device compatibility and system‐level interoperability obstructs technological iterations. Variations in operating systems and communication protocols between devices prevent real‐time data synchronization, increase the complexity of intraoperative AI use, and prevent seamless integration in orthopedic workflows [272]. Moreover, most current devices rely heavily on cloud‐based computing, in which network latency may cause delayed responses in robotic arms. In high‐precision scenarios, such delays can increase the risk of neurovascular injury, thereby severely limiting the clinical feasibility of AI‐assisted procedures [273]. Therefore, we propose that future research should focus on the development of lightweight robotic arms (e.g., bioinspired joint actuation), integrated computing architectures, and standardized cross‐vendor data communication protocols. Strengthening collaboration across industries, academia, and clinical institutions is essential for accelerating AI integration and advancing orthopedic surgical innovation.

## 4 Ethics and Regulatory Considerations

The widespread application of AI in orthopedic surgery has provided unprecedented opportunities for precision medicine and individualized treatment. However, this has raised ethical, legal, and practical concerns. These issues range from informed consent and algorithmic bias to inadequate regulatory frameworks, limited clinical acceptance, and tensions between data privacy and the need for large‐scale, multicenter, and interdisciplinary data sharing to train AI models [274, 275, 276]. Addressing these challenges requires a comprehensive engagement by researchers and policymakers through ethical, legal, and operational frameworks to ensure the responsible development and deployment of AI in orthopedic practice.

### 4.1 Ethical Issues and Societal Impact

Although AI has demonstrably enhanced the efficiency and accuracy of orthopedic surgical procedures, its implementation introduces complex ethical issues. Key concerns include patient autonomy and informed consent, algorithmic bias and fairness, accountability and liability, and the transparency and explainability of AI systems. These ethical dimensions directly influence both public trust and clinical adoption of AI tools [277].

#### 4.1.1 Informed Consent, Privacy, and Autonomy

In traditional medical practice, patient autonomy and informed consent are the foundational principles. However, the integration of AI, particularly models based on CNNs and other forms of deep learning, can complicate the consent process. These models require the collection, storage, and analysis of vast quantities of patient data, which raises significant concerns regarding data privacy and individual rights [276]. Moreover, the complexity of AI‐driven decision‐making makes it challenging for nonspecialists to fully understand the rationale behind recommendations, increasing the burden on clinicians to communicate risks effectively, and potentially diminishing patients' ability to make informed choices [278].

AI models have improved diagnostic accuracy, screening, surgical planning, intraoperative assistance, and postoperative monitoring in orthopedic surgery, such as total joint replacement. However, patients must be adequately informed of the scientific rationale behind AI‐generated recommendations and their limitations. These may include biases due to small or nonrepresentative training datasets, restricted generalizability across clinical contexts, or the use of models that have not been validated for diverse populations [279, 280].

Consequently, many patients struggle to evaluate the potential risks and uncertainties of relying on AI‐based suggestions before undergoing treatment. To address these issues, physicians must assume greater explanatory responsibilities. Clinicians are not only expected to communicate the content of AI‐generated advice but also to explain its limitations and uncertainties, for instance, whether a recommendation is derived from a small dataset or exhibits known algorithmic biases [281, 282]. The so‐called “black box” nature of many AI systems compounds these challenges. In high‐stakes procedures such as complex orthopedic surgeries, the lack of interpretability makes it difficult for clinicians to fully understand or trust AI recommendations. This further complicates accountability, as it remains unclear who bears responsibility when adverse outcomes occur, an issue that severely limits AI adoption in surgical settings [283, 284].

Additionally, AI model performance is highly dependent on the volume and diversity of the training data, making multicenter data sharing essential for producing robust and generalizable outputs. However, the need for privacy and data protection remains a major barrier. Developers must safeguard personal health information and ensure that patient rights are not compromised—including those related to employment, insurability, and interpersonal relationships. These privacy concerns often intensify the “data silo” effect, hindering collaborative model development and limiting the clinical applicability of AI in orthopedic surgery [282].

#### 4.1.2 Algorithmic Bias and Fairness

Algorithmic bias is another major ethical concern in the application of AI in orthopedic surgery. The performance and fairness of AI models depend heavily on the quality and diversity of training datasets. As demonstrated by prior studies, when AI algorithms are trained predominantly on data from specific populations, such as those defined by race or sex, their accuracy and reliability significantly decline upon application to underrepresented groups [285, 286]. For instance, a study evaluating AI models used to classify osteoporosis from chest radiographs revealed a high risk of patient selection bias, which was directly linked to the lack of diversity in the training data used by the researchers [287].

To mitigate this bias, it is essential to incorporate multicenter and demographically diverse datasets during AI model development. These datasets should include patient populations across different races, genders, and age groups to ensure broader applicability and generalizability [288]. Moreover, developing standardized datasets and advancing preprocessing techniques for handling diverse data inputs can further enhance algorithmic fairness and reduce the sensitivity to specific input variables [289, 290].

The inherent “black box” nature of many AI systems exacerbates accountability. When the decision‐making process of a model is opaque, clinicians may find it difficult to assess the scientific validity or reliability of AI recommendations. This lack of transparency, which stems from the complexity of deep learning architectures, substantially limits the adoption and integration of AI tools in orthopedic surgical practice [291, 292]. Therefore, it is critical to design AI systems with built‐in explainability to enable clinicians to interpret the basis of model decisions and challenge them when necessary.

### 4.2 Regulatory and Legal Frameworks

Despite the rapid development of AI and its expanding applications in orthopedic surgery, the corresponding regulatory and legal frameworks have not evolved at the same pace [293]. As an emerging technology, AI in healthcare poses numerous challenges in terms of visibility and legal accountability. Significant variations exist across countries and regions in the approval, implementation, and legal interpretation of AI‐powered medical devices. These discrepancies highlight the urgent need for targeted policy reforms and legal adaptations to support safe and effective AI integration [294].

#### 4.2.1 Current Regulatory Landscape for AI in Healthcare

The regulation of AI‐based medical systems is inherently complex and involves healthcare governance, technological oversight, and data privacy considerations. The rapid evolution of AI frequently outpaces existing legal structures, and regulatory standards vary widely among jurisdictions. Nevertheless, to ensure patient safety and uphold the reliability of AI tools, the continuous refinement of legal and regulatory mechanisms is essential [295, 296]. For instance, in the United States, the Food and Drug Administration has adopted a total product lifecycle approach to regulate AI‐driven medical devices. This dynamic framework includes mechanisms such as a predetermined change control plan and good machine learning practices, which aim to ensure both safety and adaptability throughout a product's lifecycle [297]. In Asia, countries such as China and Japan have begun developing specific regulatory policies for AI in healthcare. China's National Medical Products Administration has approved several AI‐enabled medical devices; however, it currently lacks clear regulatory standards addressing AI systems with adaptive or continuously learning capabilities [298].

#### 4.2.2 Legal Liability and Accountability

The issue of legal liability in AI‐driven healthcare systems has become a focal point of global debate. In orthopedic surgeries involving AI‐assisted decision‐making or robotic execution, the attribution of responsibility becomes especially complex when adverse outcomes or medical errors occur [299, 300]. Traditionally, surgeons have been held accountable for all intraoperative decisions. However, the introduction of AI has blurred these boundaries as its recommendations are generated through sophisticated algorithms and large‐scale data analytics, often beyond the expertise of the clinician. This mismatch challenges the applicability of the existing legal doctrines [301]. For example, in robot‐assisted orthopedic procedures, AI may recommend the prosthesis position or bone‐cutting trajectory, and the surgeon executes the operation based on these suggestions. If the patient later experiences serious complications such as prosthetic loosening or nerve injury, questions arise: Was it a surgical error, a flaw in the AI algorithm, or an issue with the underlying data? Current legal frameworks struggle to address the multifactorial causality in technologically mediated care.

The maturity of the regulatory and legal frameworks will directly influence the broader adoption and trustworthiness of AI in orthopedic surgery. As technology continues to evolve, traditional regulatory approaches to medical devices may no longer suffice for AI systems with dynamic and autonomous behaviors. Future legal paradigms must adopt flexible and responsive models capable of balancing innovation with safety. Such frameworks will be vital not only for protecting patient rights, but also for promoting the sustained advancement of AI technologies in orthopedic clinical practice.

## 5 Future Directions

AI in orthopedic surgery is progressively evolving from an auxiliary tool to a full‐process intelligent system. Future developments will primarily revolve around four key trends: technical autonomy, data convergence, interdisciplinary integration, and globalized applications. Through continuous advancements in algorithm development, device innovation, and the refinement of ethical and regulatory frameworks, AI is poised to strongly drive the realization of the “precision medicine” goal. Among these, cross‐disciplinary collaboration and global cooperation have emerged as critical factors for overcoming technical bottlenecks, whereas cost control and enhancing physician awareness represent the core pathways for promoting universal healthcare accessibility. The authors posit that, as AI technologies continue to mature, they will persistently reshape orthopedic diagnostic and therapeutic paradigms, significantly elevate the precision of orthopedic procedures, and thereby deliver safer, more efficient, and personalized treatment plans for patients.

### 5.1 Technological Frontiers and Breakthroughs

In the future, technological advancements in AI for orthopedic surgery will be centered on higher degrees of autonomy and integration of emerging technologies [302]. Several key directions are poised to become major drivers of innovation in this field.

#### 5.1.1 Autonomous Surgical Robotics

Currently, AI applications in orthopedic surgery are predominantly assistive in nature, with the final decisions made by the surgeon. However, with the ongoing advances in AI, particularly in self‐learning algorithms, and the development of modular, intelligent, lightweight, and highly integrated devices, fully autonomous surgical robotic systems are expected to become a major focus of research. These next‐generation systems combine adaptive learning algorithms with real‐time environmental sensing to dynamically tailor operative paths and strategies for individual patient profiles. Such capabilities could not only reduce the clinical workload and healthcare costs, but also significantly enhance surgical precision and efficiency [303, 304]. For example, computer‐assisted robotic systems for fracture reduction have already begun to demonstrate semi‐autonomous capabilities. Recent developments in fracture reduction robotics show promise for automating complex orthopedic maneuvers while providing technical support to surgeons, effectively enhancing their intraoperative performance and leveraging robotic potential for procedural consistency and reproducibility [305].

#### 5.1.2 Multimodal Data Integration

Orthopedic surgery relies on the integration of various data types, such as imaging, biomechanical parameters, and biological signals, to tailor surgical strategies according to individual patient conditions. The goal is to maximize the therapeutic outcomes while minimizing complications. The rapid evolution of AI is reshaping the surgical paradigm. Through deep neural networks and multimodal medical data fusion, AI systems have become increasingly capable of providing intelligent, full‐cycle support for orthopedic procedures, from preoperative planning and intraoperative navigation to postoperative rehabilitation. Several AI models have been shown to reduce the intraoperative decision‐making time and minimize unnecessary surgical trauma [306, 307, 308].

Moreover, multimodal integration offers significant advantages for enhancing surgical precision. In clinical settings, the fusion of imaging data (e.g., CT, MRI, and intraoperative ultrasound) with physiological metrics (e.g., bone density and muscle tension) provides AI systems with a more comprehensive basis for decision‐making [309]. For instance, combining 3D CT reconstructions with biomechanical simulations allows AI to predict how different screw diameters and trajectories affect spinal stability, thereby optimizing implant strategies. This data‐driven planning approach has already demonstrated an 18% reduction in the postoperative complication rates in complex spinal deformity surgeries [310].

We anticipate that future AI systems will increasingly leverage edge computing and cloud‐based platforms to provide real‐time analysis and processing of multimodal data. This will not only facilitate optimized surgical path planning but also enable continuous intraoperative monitoring and dynamic decision support. Particularly in complex surgeries, real‐time multimodal data integration can significantly reduce intraoperative risks and improve surgical outcomes.

#### 5.1.3 Integration of Emerging Technologies

The deep integration of AI with robotic technologies has accelerated the evolution of orthopedic surgery toward autonomous systems. At Stanford University, researchers developed a cognitive surgical robotics system that combines computer vision with reinforcement learning algorithms to dynamically analyze intraoperative imaging and adjust surgical trajectories in real time. In a clinical study involving 120 spinal surgeries, the system reduced pedicle screw placement time by 42% while maintaining a complication rate below 0.8% [311]. Notably, AI‐driven robotic systems are capable of continuously learning from surgeons’ operational patterns, enabling the automatic generation of personalized surgical strategies. A study from Johns Hopkins University demonstrated that such intelligent systems could improve the surgical performance of junior surgeons compared with that of experienced specialists [312]. In parallel, emerging technologies, such as quantum computing and digital twin modeling, are opening new avenues for AI‐enhanced orthopedic surgery.

Quantum computing offers transformative potential for orthopedic biomechanical research. Conventional computing systems are often constrained by time and processing power when handling large data sets or complex algorithms. By contrast, quantum computing accelerates the training of deep learning models through parallel computation, enabling more efficient biomechanical feature extraction and surgical plan generation [313]. Quantum machine learning algorithms can optimize the identification of surgical parameters and improve personalized treatment planning. Second, quantum‐encryption techniques promise to enhance data security during transmission and analysis. Finally, the convergence of quantum computing with surgical robotics can facilitate real‐time biomechanical feedback during surgery, allowing dynamic procedural adjustments. These innovations not only improve surgical precision and safety but also redefine surgical workflows in the era of individualized medicine.

Digital twin technology, which integrates anatomical and physiological data to simulate patient‐specific scenarios, represents another frontier in orthopedic surgery. By generating real‐time, continuously updated virtual models of the patient, digital twins enable predictive modeling of surgical outcomes and optimization of treatment strategies [314, 315]. This approach holds considerable promise in improving patient prognosis and enhancing surgical precision. However, it also introduces several technical and ethical challenges, including the need for large‐scale datasets, system integration complexities, and concerns regarding data privacy and legal accountability. Future research must address these challenges to enable seamless integration of digital twin systems with AI and deep learning architectures [316, 317].

### 5.2 Interdisciplinary Collaboration and Global Dissemination

Advancement and widespread adoption of AI in orthopedic surgery are inseparable from interdisciplinary collaboration and international cooperation. As a technology‐intensive specialty, orthopedic surgery requires the deep integration of AI with fields such as computer science, materials science, and biomedical engineering [318]. In parallel, the establishment of global data‐sharing frameworks and standardized dissemination strategies is essential for scaling AI applications, enabling a transition toward more intelligent and precise orthopedic interventions [319].

#### 5.2.1 Interdisciplinary Integration

The application of AI in orthopedic surgery has greatly benefited from continuous advances in computer science, materials science, and biomedical engineering [320, 321]. Strengthening core computational technologies, such as deep learning, reinforcement learning, and computer vision, is essential for enhancing the ability of AI to process complex biomedical data and generate accurate treatment recommendations. The integration of AI with mechanical engineering is vital for improving the performance of orthopedic surgical robots and expanding their clinical utility [322, 323]. Establishing interdisciplinary laboratories that unite clinicians, engineers, and data scientists will accelerate innovation and support the rapid development of AI‐driven solutions.

AI has revolutionized orthopedic implant design in the field of biomedical engineering. Machine learning algorithms can analyze patient‐specific features such as bone mineral density, mobility patterns, and biomechanical characteristics to develop highly personalized implants [324, 325]. Smart implants embedded with biosensors may offer real‐time monitoring and dynamic functional adjustment, thereby significantly improving therapeutic outcomes.

#### 5.2.2 Global Collaboration and Data Sharing

The long‐term advancement of AI in orthopedic surgery depends on the global sharing of healthcare data. Building multinational collaborative networks and standardized data platforms is crucial to improve the generalizability of AI models and expand access to high‐quality orthopedic care [326]. Research has shown that incorporating data from diverse populations across races, sexes, and age groups can reduce algorithmic bias and enhance predictive performance. However, data privacy regulations and ethical compliance remain major barriers to global data exchange [327, 328].

Federated learning has emerged as a promising approach for addressing these challenges. This enables institutions to train AI models collaboratively without sharing raw data, thereby balancing data utility and privacy protection [329]. Notably, this technique has been applied to integrate data from 20 international medical centers to predict oxygen requirements, ICU admissions, and mortality risks in COVID‐19 patients with—significantly improving model accuracy and generalizability [330].

In future, optimized federated learning frameworks will enable more robust multinational collaborations, enriching the training datasets available for orthopedic AI models. This will not only promote global innovation in surgical technologies but also offer new strategies for managing complex diseases across regions. Furthermore, the development of global health data alliances combined with blockchain‐based platforms may ensure data security and integrity, further enhancing the performance and trustworthiness of AI applications in orthopedic surgery.

#### 5.2.3 Technology Adoption and Implementation Barriers

Despite its immense potential, the widespread adoption of AI in orthopedic surgery still faces multiple challenges. AI‐powered tools and data systems are rapidly transforming the surgical landscape from preoperative planning and intraoperative navigation to postoperative monitoring and operational logistics. Wearable devices, smart sensors, robot‐assisted systems, AI‐driven imaging, and 3D modeling technologies have significantly expanded the role of AI across the orthopedic care continuum [331, 332]. However, two major barriers remain, namely, high technological costs and physician acceptance. Many primary healthcare institutions, particularly in resource‐limited settings, lack the financial capacity to procure and maintain AI‐based surgical equipment. Additionally, clinicians’ trust in and proficiency in AI tools directly affect their clinical effectiveness. In complex cases, surgeons may prefer to rely on personal experience rather than AI‐generated recommendations [244, 333].

As the cost of AI technology decreases and awareness among healthcare professionals improves, AI applications are likely to be adopted worldwide. This shift will not only help address healthcare resource disparities, but also drive a new wave of innovation and service enhancement in orthopedic care.

## 6 Conclusion

AI has driven a transformative shift in orthopedic surgery. Through its intelligent applications in preoperative planning, intraoperative navigation, and postoperative rehabilitation, AI has significantly improved the precision and efficiency of surgical care, while reducing the overall cost of clinical practice. The integration of multimodal data with deep learning models has enabled the development of highly personalized treatment strategies for complex cases. In parallel, the rapid advancement of intelligent surgical robotics and digital twin technologies has not only optimized the surgical pathway design but also provided scientific guidance for postoperative recovery planning. The combined deployment of these technologies is reshaping orthopedic surgical paradigms and ushering in a new era of intelligent personalized orthopedic care.

Nonetheless, the widespread implementation of AI in orthopedic surgery presents considerable challenges. On the algorithmic front, there remains substantial room for improvement in both the model performance and robotic device capabilities. Limitations related to data bias and insufficient diversity restrict the generalizability of the model. Furthermore, hardware development is still in its infancy, which constrains the real‐world applications of AI‐enhanced systems. The “black‐box” nature of many AI models also impedes clinical trust, particularly in high‐stakes, complex procedures, where the lack of transparency and interpretability hinders broad clinical acceptance.

From ethical and regulatory perspectives, concerns about data privacy and legal accountability remain significant barriers to adoption. In multicenter collaborations and data‐sharing initiatives, achieving a balance between efficient cooperation and robust privacy protection remains an unresolved challenge.

The evolution of AI in orthopedic surgery is driven by technological optimization, device innovation, and cross‐disciplinary collaboration. Advances in multimodal data integration and privacy‐preserving approaches such as federated learning will facilitate more efficient data sharing and model training. The integration of quantum computing, smart materials, and digital twin systems will open new frontiers in the diagnosis and treatment of complex orthopedic conditions. The deep convergence of orthopedics, computer science, and biomedical engineering will further accelerate the translation of AI technologies from research to clinical applications, enabling more precise and efficient surgical care.

To fully realize the potential of AI in orthopedic surgery, future research should prioritize several key areas. First, the development of more robust and generalizable algorithms and robotic systems will enhance their functional performance and broaden their clinical applicability. Second, improving model transparency and explainability will help clinicians understand the rationale behind AI‐driven decisions better, thereby strengthening trust. Third, expanding professional training for physicians and technical staff will improve their acceptance and proficiency in using AI tools. Finally, building international, interdisciplinary collaborations and secure data‐sharing platforms, supported by federated learning and sound ethical and legal frameworks, will lay the groundwork for large‐scale, equitable deployment. Collectively, these efforts will propel orthopedic surgery toward a more intelligent and personalized future.

## Author Contributions

Fei Han and Xiao Huang wrote the manuscript and summarized the table contents. Fei Han and Da Wei Zhang created the images. Xin Wang and Fei Han designed the structure and organized the manuscript. Da Wei Zhang, Yong Feng Chen, Chuang Lu, Shasha Li, and Lu Lu reviewed and revised the structure, ideas, and scientific validity of the manuscript. All authors have read and approved the final manuscript.

## Ethics Statement

The authors have nothing to report.

## Conflicts of Interest

The authors declare no conflicts of interest.

## Data Availability

Data availability is not applicable to this article as no new data were created or analyzed in this study.
